# *DAMMIF*, a program for rapid *ab-initio* shape determination in small-angle scattering

**DOI:** 10.1107/S0021889809000338

**Published:** 2009-01-24

**Authors:** Daniel Franke, Dmitri I. Svergun

**Affiliations:** a European Molecular Biology Laboratory, Hamburg Outstation Notkestrasse 85, 22603 Hamburg, Germany; b Institute of Crystallography, 117333 Moscow, Russian Federation

**Keywords:** computer programs, *DAMMIF*, *DAMMIN*, small-angle scattering, particle shape determination

## Abstract

*DAMMIF*, a revised implementation of the *ab*-*initio* shape-determination program *DAMMIN* for small-angle scattering data, is presented. The program was fully rewritten, and its algorithm was optimized for speed of execution and modified to avoid limitations due to the finite search volume. Symmetry and anisometry constraints can be imposed on the particle shape, similar to *DAMMIN*. In equivalent conditions, *DAMMIF* is 25–40 times faster than *DAMMIN* on a single CPU. The possibility to utilize multiple CPUs is added to *DAMMIF*. The application is available in binary form for major platforms.

## Abstract 2

*DAMMIF*, an enhanced and significantly faster implementation of the *ab*-*initio* shape-determination program *DAMMIN* for small-angle scattering data, is presented.

## 1. Introduction   

Small-angle scattering (SAS) of X-rays and neutrons is a fundamental tool in the study of the nanostructure of matter, including disordered systems and solutions (Feigin & Svergun, 1987 ▸). In a scattering experiment, the specimen (*e.g.* particles of nanometre-scale size floating in solution or embedded in a bulk matrix) is exposed to X-rays or neutrons, and the scattered intensity 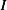 is recorded. For disordered systems, the random positions and orientations of particles lead to an isotropic intensity distribution 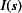, which depends on the modulus of momentum transfer 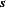 (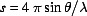, where 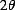 is the angle between the incident and scattered radiation, and 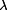 is the wavelength). If the system contains identical non-interacting particles, for example for monodisperse dilute solutions of purified biological macromolecules, 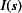 is proportional to the scattering from a single particle averaged over all orientations. This allows one to obtain information about the overall shape and internal structure of particles at a resolution of 1–2 nm (Feigin & Svergun, 1987 ▸; Svergun & Koch, 2003 ▸).

Recent progress in instrumentation and development of data analysis methods (Svergun & Koch, 2003 ▸; Petoukhov *et al.*, 2007 ▸) has significantly enhanced the resolution and reliability of the models provided by SAS. A number of novel approaches have been proposed to analyse the scattering data from monodisperse systems in terms of three-dimensional models [see Petoukhov *et al.* (2007 ▸) for a review]; these advances have significantly increased the popularity of SAS in the study of biopolymers in solution. Among these methods, *ab*-*initio* shape determination techniques are especially important: first, they do not require *a-priori* information about the particle, and second, they are applicable also for moderately polydisperse (nonbiological) systems, allowing one to retrieve the overall averaged shape over the ensemble (Shtykova *et al.*, 2003 ▸, 2007 ▸).

The aim of *ab-initio* analysis of SAS data is to recover the three-dimensional structure from the one-dimensional scattering pattern, and unique reconstruction is only possible in the trivial case of a spherical particle. In shape determination, one represents the particle by homogeneous models to constrain the solution and reduce the ambiguity of the reconstruction. This simplification usually is justified in the analysis of the low-angle scattering patterns from single-component particles. In all *ab-initio* methods, particle shape is represented in real space by a parametric model, and the parameters of the model are altered so as to minimize the difference between the computed scattering of the model and the experimental data. A number of methods and alternative programs exist, which differ primarily in the way the shape is represented. In the first general *ab-initio* approach (Stuhrmann, 1970 ▸), an angular envelope function was implemented in the program *Sasha* (Svergun *et al.*, 1996 ▸), which was limited to globular particles without significant internal cavities. More detailed models are obtained by representing the particle by finite volume elements, thus allowing internal cavities to be accounted for. Using beads to model the scattering object, which was first proposed by Chacon *et al.* (1998 ▸) and implemented in the program *DALAI_GA*, a search volume is filled by densely packed small spheres (also referred to as dummy atoms), which are assigned either to the particle or to the solvent. Starting from a random assignment, a Monte Carlo search, for example a genetic algorithm in *DALAI_GA* or simulated annealing (SA) in *DAMMIN* (Svergun, 1999 ▸), is employed to find a model that fits the data. A similar approach was implemented in the Give’n’Take procedure of *SAXS3D* (Walther *et al.*, 2000 ▸), which runs on a grid of unlimited size. Heller *et al.* (2002 ▸) developed a program *SASMODEL*, representing the particle by a collection of interconnected ellipsoids.

*Ab-initio* methods have been proven to reliably reconstruct the low-resolution shape in numerous tests and practical studies, and they now belong to routine tools in SAS data analysis. Since little or no information has to be specified by the user in most cases, these methods are currently being incorporated into high-throughput automated data analysis pipelines (Petoukhov *et al.*, 2007 ▸). The extensive use of the shape determination programs, including large scale studies, makes the speed of reconstruction a rather important issue. The Monte Carlo-based algorithms usually require millions of random models to be screened and are thus time consuming. Moreover, given that different shapes are obtained starting from different initial random models, often ten or more *ab-initio* runs need to be performed and averaged to assess the uniqueness of the solution and to reveal the most persistent shape features (Volkov & Svergun, 2003 ▸). Presently, most shape determination programs require hours of CPU time for a single run on a typical Windows or Linux PC; clearly this time needs to be reduced to the order of minutes or less.

This paper describes a new implementation of *DAMMIN* (Svergun, 1999 ▸), one of the most popular shape determination programs publicly available. The program, called *DAMMIF* (where ‘F’ denotes fast), has been completely rewritten in object-oriented code and the algorithm has been optimized for speed and efficiency. The algorithm was further improved in an attempt to avoid artifacts caused by the limited search volume. This was achieved by replacing the closed with an unlimited and growing search volume . A version of *DAMMIF* optimized to make use of multiple CPUs is also available. Furthermore, the implementation of *DAMMIF*, like *DAMMIN*, features options to account for symmetry and anisometry in the modelling if the relevant information is available.

## 2. *DAMMIN* algorithm   

In this section, we outline the major features of *DAMMIN* that are important for an understanding of the *DAMMIF* algorithm. The reader is referred to the original publication (Svergun, 1999 ▸) for further details.

In the original version of *DAMMIN*, a search volume (usually a sphere with radius 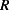 equal to half the maximum particle size 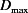) is filled with densely packed small spheres of radius 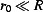. Each sphere may belong either to the particle (index = 1) or to the solvent (index = 0). The shape of this dummy atom model (DAM) is described by a binary configuration vector 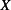 of length 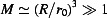. The scattering intensity from the configuration 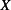 is calculated as 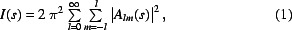
where the partial scattering amplitudes are 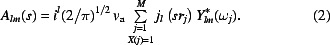


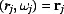
 are their polar coordinates, 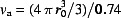 is the displaced volume per dummy atom, 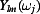 are the corresponding spherical harmonics and 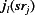 denote spherical Bessel functions. The function 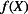 to be minimized has the form 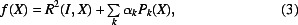
where the first term on the right-hand side is the discrepancy between the experimental and calculated data, and the second term summarizes penalties as listed in Table 1 ▸ weighted by appropriate factors.

The result after running the application is a compact interconnected DAM that fits the experimental data. If information about the particle symmetry is available, it is taken into account as a hard constraint by changing all the symmetrical dummy atoms simultaneously. *A-priori* information about the particle anisometry can also be taken into account.

The spherical harmonics expansion using equations (1) and (2) is computationally superior to the standard Debye (1915 ▸) formula, which is usually employed to compute the scattering from bead models. Moreover, only a single dummy atom is changed at each move and hence only a single summand in equation (2) must be updated to recalculate the partial amplitudes. This accelerates *DAMMIN* significantly, but still, as millions of function evaluations are required, a typical refinement takes about 2–3 CPU hours on an average PC for a DAM containing a few thousands spheres.

## 3. *DAMMIF* implementation   

Similar to *DAMMIN*, *DAMMIF* uses the scattering pattern processed by the program *GNOM* (Svergun, 1992 ▸); *DAMMIF* also follows the general algorithm of *DAMMIN*.

The program was, however, completely rewritten with the main aim of speeding up the operation. Major algorithmic changes in *DAMMIF* are described in the following sections.

### 3.1. Bead selection   

**Figure 1:**
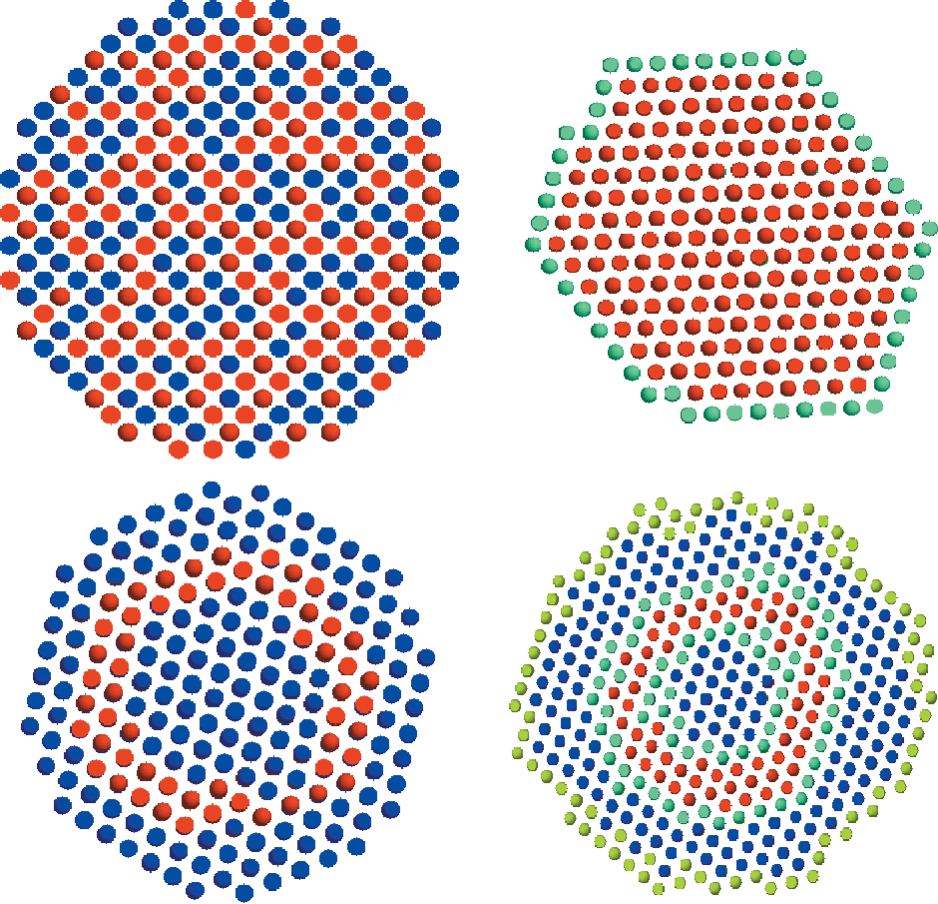
Cross sections of dummy atom models of *DAMMIN* (left) and *DAMMIF* (right). The top row shows initial models (randomized and proto-particle) and the bottom row the final models by the two programs. The different colours indicate particle (red) and solvent (turquoise, blue and green) states of the dummy atoms. In *DAMMIF*, only red and turquoise beads are subject to phase changes; *DAMMIN* generally allows phase transitions anywhere in the search volume. Green solvent beads indicate the current, extensible, border of *DAMMIF*’s mapped area (all visible beads).

A very important constraint for low-resolution *ab-initio* modelling is that in the final model all beads representing the particle must be interconnected to form a single body. Implementation of this condition is different between *DAMMIN* and *DAMMIF*. Fig. 1 ▸ shows examples of the cross sections through the initial and final bead models (top and bottom row, respectively) of *DAMMIN* (left) and *DAMMIF* (right). The beads are colour coded as belonging to the particle (red) and solvent (turquoise, blue, green) phases. Turquoise and green beads differ from blue ones only in that the former are relevant for the bead-selection algorithm described in the next paragraph and the latter for the unlimited search volume as described in the next section.

For each annealing step, *DAMMIN* and *DAMMIF* select a bead completely at random. *DAMMIN* updates the simulated scattering, computes the fit and penalizes possible disconnectivity of the particle beads before deciding whether to accept or reject the change. There, the disconnectivity is defined by the length of the longest graph (ensemble of beads, where each pair can be connected by moving through the beads touching each other in the grid), which is a CPU-intense operation. *DAMMIF* tests connectivity first and rejects disconnected models before launching into the time consuming process of updating the scattering amplitudes. The latter are computed if and only if a particle bead (red) or an adjacent bead (turquoise) is selected (Fig. 1 ▸); otherwise the step is cancelled and execution is resumed with the next step. A summary of the set of rules used to decide about the connectivity of models is given in Table 2 ▸.

### 3.2. Unlimited search volume   

In *DAMMIN*, the search volume is configurable at runtime but fixed throughout the search procedure. The search volume is filled with densely packed dummy atoms before SA begins. Limiting the volume may be a useful feature for shape reconstruction (in particular, nonspherical search volumes can be employed to account for additional information about the shape, if available). However, in some cases, especially for very anisometric particles, a restricted search volume may lead to artifacts. Indeed, the bead representing the particle is obviously prevented from protruding outside the border of the search volume. If, during the reconstruction, the particle is formed close to the border, the search space becomes anisotropic, possibly leading to unwanted border effects like artificial bending. To avoid such effects, the algorithm of *DAMMIF* was modified, allowing for the search in a variable volume which is extended as necessary during the SA procedure. In the following, we shall mostly refer to this unlimited *DAMMIF*, but a bounded-volume version is also available on request.

Unlike *DAMMIN*, which fully randomizes the closed search volume on start-up (Fig. 1 ▸, top left panel), *DAMMIF* starts from an isometric object with the radius of gyration (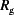) matching the experimentally obtained one (Fig. 1 ▸, top right panel). This proto-particle (red) is constructed by adding successive layers of beads until the desired 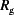 is reached. The polyhedral appearance of the starting model as shown in Fig. 1 ▸ is subject to the hexagonal packing of beads – it should be noted that the shape of the initial model has practically no influence on the reconstruction. The starting shape is then covered by a single layer of solvent beads, shown in green. The green colour implies that, if such a bead is selected for phase transition, potentially missing neighbours are added to the search volume. To accomplish this, the coordinates of the neighbours are computed and looked up in the list of available beads. If a neighbour is missing, its coordinates are added as a new bead of solvent phase to the said list. To avoid runtime penalties due to linear searches on ever-growing lists, beads are stored in multidimensional binary search trees (Bentley, 1975 ▸), which are also known as 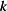
d-trees. Furthermore, amplitudes of newly created dummy atoms are lazily evaluated, *i.e.* they are not computed until they contribute to the particle scattering for the first time. Although lazily computed, once available partial amplitudes are stored in a cache for later re-use.

Adding neighbours as described ensures that beads in the particle phase (index = 1) are always surrounded by beads in the solvent phase (index = 0). Thus, the algorithm may traverse a potential, but not yet mapped, search volume. This was not possible in *DAMMIN*, where the closed search volume may have blocked the annealing algorithm from potentially better results.

### 3.3. Penalties   

Penalties impose a set of rules on the dummy atom model to modify its likelihood of being accepted by the SA selection rule [equation (3), right-hand sum]. Hence, penalties are used to guide the annealing process. In general terms, the bead-selection algorithm presented above implements an implicit penalty. Owing to the improved rejection of disconnected models (Fig. 1 ▸ and Table 2 ▸), the likelihood of accepting a disconnected model constantly equals zero.

Table 1 ▸ summarizes the different sets of penalties implemented in *DAMMIN* and *DAMMIF*. In *DAMMIF*, the peripheral penalty was dropped as there is no more outer boundary to limit particle growth. Furthermore, the disconnectivity penalty became implied as a result of improved rejection of unwanted disconnected models. Instead, centre and 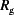 penalties were introduced. The role of the centre penalty is to keep the particle within the already mapped space, to prevent needless extension (and thus calculation) of the search volume, and the 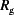 penalty ensures a model of appropriate size. Looseness and anisometry penalties are implemented by both applications.

### 3.4. Parallelization   

**Figure 2:**
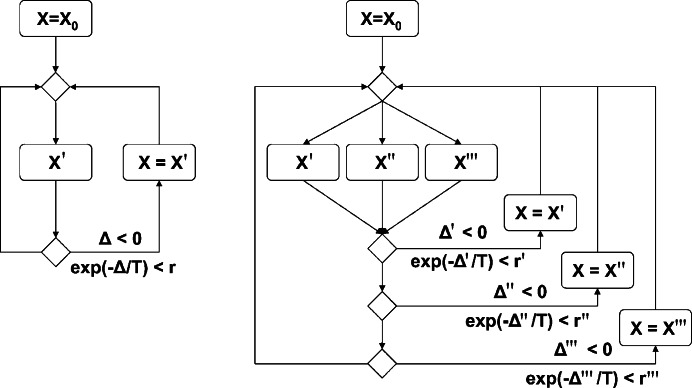
SA algorithm as implemented in *DAMMIN* (left) and *DAMMIF* (right). An initial starting model 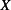 is refined to yield the best possible fit to the experimental data. In *DAMMIN*, only one neighbouring model 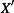 is taken into account at a time. If multiple cores or CPUs are available, it is possible to *prefetch* multiple models in parallel, here shown as 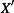, 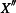, 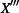. Each prefetched model is then examined and either accepted or rejected, according to the rules of SA.

In *DAMMIN*, the SA algorithm is implemented as follows (Fig. 2 ▸, left-hand side):

(i) Start from a random configuration 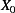 at a high temperature 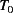 [*e.g.* 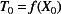].

(ii) Flip the index of a randomly selected dummy atom to obtain configuration 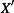 and compute 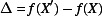.

(iii) If 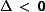, move to 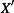; if 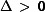, move to 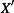 with probability 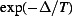. Repeat step (ii) from 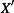 (if accepted) or from 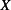.

(iv) Hold 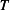 constant for 100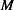 reconfigurations or 10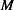 successful reconfigurations, whichever comes first, then cool the system 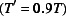. Continue cooling until no improvement in 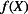 is observed.

It can easily be seen that the longer the algorithm proceeds, the less likely a successful reconfiguration becomes. As multi-core and multi-CPU systems are becoming more readily available, *DAMMIF* also makes use of these resources. To further speed up *ab-initio* modelling, *DAMMIF* employs OpenMP, a framework for shared memory parallelization (Dagum & Menon, 1998 ▸). To exploit the properties of SA as described above, a simple prefetch and branch prediction scheme was implemented (Fig. 2 ▸, right-hand side). Instead of a single neighbouring model 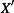 as in *DAMMIN*, *DAMMIF* computes multiple models 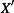, 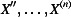 in parallel (prefetch). Of these it is likely that most, if not all, will be rejected in later temperature steps. Hence, computing many neighbouring models ahead of time corresponds to a negative branch prediction.

## 4. Quality of reconstruction and practical aspects   

**Figure 3:**
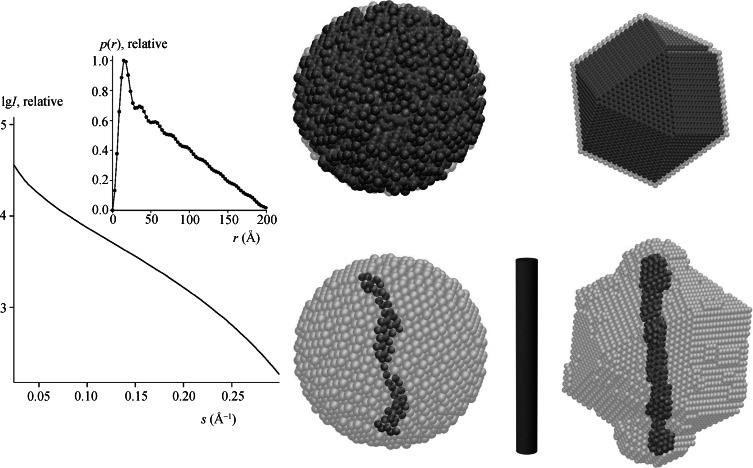
Reconstruction of a cylindrical particle with radius 10 Å and height 200 Å (bottom centre) from its simulated scattering pattern presented on the left-hand side [relative intensity *I versus* inverse ångströms; the distance distribution function *p*(*r*) computed by *GNOM* is displayed in the insert]. The starting (top row) and final (bottom row) models from *DAMMIN* and *DAMMIF* are displayed in the middle and right panels, respectively. *DAMMIN* ran in a slow mode inside the spherical search volume (packing radius 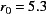 Å, CPU time used 246 min). For *DAMMIF*, the value of 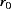 was 3.0 Å and the run on the same single processor took 8 min.

Extensive tests on simulated and experimental data showed that the models provided by *DAMMIF* are comparable to those of *DAMMIN* and the quality of reconstruction is compatible with that presented by Svergun (1999 ▸) and Volkov & Svergun (2003 ▸). For highly anisometric particles, the models provided by *DAMMIF* may be more accurate thanks to the absence of border effects. A comparison of model reconstructions by *DAMMIN* and *DAMMIF* of a cylindrical particle with radius 10 Å and height 200 Å is illustrated in Fig. 3 ▸.

Of course, *DAMMIF*, similar to *DAMMIN* and other shape determination programs, is not applicable to heterogeneous systems like mixtures or unfolded proteins. For the analysis of higher-resolution data from small (less than 30 kDa) proteins, where the contribution from the internal structure is essential, other programs like *GASBOR* (Svergun *et al.*, 2001 ▸) may be more appropriate for *ab-initio* analysis than the shape determination algorithms.

The 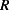 factor 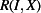 [see equation (3)] of the obtained *DAMMIF* model, which is provided to the user in the log file and in the PDB-type file (Protein Data Bank; Berman *et al.*, 2000 ▸) containing the final solution, permits one to rapidly assess the quality of the reconstruction. Usually, 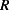 factors exceeding 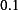 indicate poor fits and therefore point to incorrect assumptions about the object under study. It is further extremely important to analyse the uniqueness of the reconstruction, similar to *DAMMIN*, by comparing and averaging  multiple individual runs, *e.g.* using the program *DAMAVER* (Volkov & Svergun, 2003 ▸). The improved speed of *DAMMIF* allows the user to perform these analyses in a much shorter time.

## 5. Conclusions   

Here we present *DAMMIF*, an advanced implementation of the popular *ab-initio* modelling program *DAMMIN* (Svergun, 1999 ▸). Table 3 ▸ summarizes the differences between these two implementations: most notable is a reduction of the average runtime by a factor of 25–40, depending amongst other factors on the number of dummy atoms in the search model. Furthermore, a pre-defined search volume that limits mapping of possible solutions was replaced by an unlimited, adapting search space.

Additional constraints such as particle symmetry and anisometry are available in *DAMMIF* as they are in *DAMMIN* (*i.e.* as a hard constraint) – except for some higher symmetries listed in Table 3 ▸ where *DAMMIN* itself is very fast. As an additional option, *DAMMIF* is able to output pseudo-chains in PDB-format files to make them more suitable for submission to the PDB.

In the present implementation of *DAMMIF*, most of the reduction in runtime is due to algorithmic improvements, such as differences in bead selection, and not due to parallelization (Fig. 2 ▸). Because *DAMMIF* extensively employs look-up tables and thus uses more RAM, the memory-transfer overhead significantly reduces the gain from the use of multiple CPUs. This will be investigated and, if possible, improvements will be added to later versions of the application.

Further work is also in progress to implement the prefetch strategy (Fig. 2 ▸), to parallelize other CPU-intensive programs from the *ATSAS* package (Konarev *et al.*, 2006 ▸) that employ SA for model building in small-angle scattering.

### 5.1. Availability   

*DAMMIF* is available in binary format for major platforms (Windows, Linux, MacOSX) from the *ATSAS* web page (http://www.embl-hamburg.de/ExternalInfo/Research/Sax/software.html).

## Figures and Tables

**Table 1:** Penalties as implemented by *DAMMIN* and *DAMMIF* by function and type Explicit penalties are configurable and may be disabled; implicit penalties are enforced and may not be disabled.

		Function				*DAMMIN*	*DAMMIF*
Peripheral penalty (gradually decreasing)		Keeps the particle beads close to the origin at high temperatures				Explicit	
Disconnectivity penalty		Ensures that the model is interconnected				Explicit	Implicit
Looseness penalty		Ensures that the model is compact				Explicit	Explicit
Anisometry penalty (with symmetries only)		Specifies whether the model should be oblate or prolate				Explicit	Explicit
Centre/ 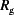 penalty		Keeps the centre of mass of the model close to the origin					Explicit

**Table:** Following these rules, early rejection can be based on the number of graphs before and after the proposed change. In particular, if the change leads to two or more graphs in a model without symmetry, the model becomes disconnected.Case 1: bead 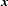 of solvent phase was selected to switch to particle.

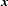 has …	neighbours in particle phase, then …
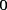	create a new graph, add 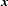
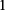	add 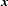 to the graph the neighbour belongs to
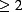	merge all graphs the neighbours belong to, add 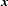

**Table:** Case 2: bead 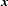 of particle phase was selected to switch to solvent.

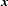 has …	neighbours in particle phase, then …
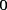	find and remove the graph built by 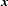
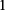	find the graph 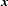 belongs to, remove 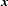
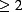	find the graph 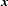 belongs to, split into two or more graphs if 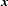 is an articulation point, remove 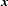

**Table 3:** Summary of differences between implementations of *DAMMIN* and *DAMMIF*

	*DAMMIN*	*DAMMIF*
Expected runtime, fast mode†	15min	30s
Expected runtime, slow mode†	24h	1h
Memory usage, slow mode†	10 MB	100 MB
Search volume	Closed	Unlimited
Particle symmetry constraints	Yes	Yes‡
Particle anisometry constraints	Yes	Yes
Model chaining	No	Yes§
Parallelization	No	Yes
Platforms	Windows, Linux	Windows, Linux
Implementation language	Fortran 77	Fortran 95

† The CPU wall clock times for a run on a typical PC without symmetry restrictions are given. Fast and slow mode: packing radius corresponds to *ca* 2000 and *ca* 10000 dummy atoms, respectively, in a sphere with radius 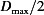.

‡ Same as in *DAMMIN*, but the space groups *P*23 and *P*432 and icosahedral symmetry are not implemented.

§ Optionally, sorts the dummy atoms in the output file to form pseudo-chains.
